# Supplementary material for: Knowledge, attitudes, and practices of cardiac rehabilitation and barriers to referral among cardiologists in Saudi Arabia: A cross-sectional survey
Source: PLoS One. 2025 May 16;20(5):e0323694. doi: 10.1371/journal.pone.0323694 (PMC12083838; doi:10.1371/journal.pone.0323694)
Supplement: S1 Table — (DOCX) [file pone.0323694.s001.docx]

## **Supplementary Table 1: Knowledge Univariate Linear Regression n=106**

|  | **Estimate** | **Std. Error** | **t value** | **P** |
| --- | --- | --- | --- | --- |
| **Sex: Male compared to Female** | 1.066 | 0.430 | 2.477 | 0.015 * |
| **Age category**  **<35 compared to** |  |  |  |  |
| *35-40* | -0.271 | 0.441 | -0.614 | 0.541 |
| *41-55* | 0.496 | 0.425 | 1.165 | 0.247 |
| *56-65* | 0.450 | 0.551 | 0.817 | 0.416 |
| *>65* | 0.200 | 1.469 | 0.136 | 0.892 |
| **Nationality**  **Non-Saudi Arabian compared to Saudi Arabian** | -0.204 | 0.285 | -0.716 | 0.475 |
| **Specialty Level**  **Fellow compared to** |  |  |  |  |
| *Consultant* | 0.780 | 0.394 | 1.982 | 0.050* |
| *Specialist* | 0.415 | 0.482 | 0.859 | 0.392 |
| **Education Location**  **Saudi Arabia compared to** |  |  |  |  |
| *Middle East/Africa Not Saudi Arabia* | -0.491 | 0.476 | -1.032 | 0.305 |
| *USA* | -0.279 | 0.573 | -0.487 | 0.627 |
| *Canada* | 0.121 | 0.411 | 0.293 | 0.770 |
| *Europe* | 0.371 | 0.525 | 0.706 | 0.482 |
| *South Asia* | 0.637 | 0.879 | 0.725 | 0.470 |
| *Multiple* | -0.029 | 0.879 | -0.033 | 0.973 |
| *Unknown* | -0.363 | 0.452 | -0.802 | 0.424 |
| **Attitude 1: Do you think that a patient who is stable post percutaneous coronary intervention procedure should be enrolled into a cardiac rehabilitation program?**  **Likert scale 1 to 5** | 0.431 | 0.170 | 2.535 | 0.013* |
| **Attitude 2: Do you think that cardiac rehabilitation in Saudi Arabia is effective?**  **Likert scale 1 to 5** | 0.196 | 0.129 | 1.515 | 0.133 |
| **Attitude 3: Do you think that your patients’ outcomes improved when they are enrolled in cardiac rehabilitation?**  **Likert scale 1 to 5** | 0.703 | 0.207 | 3.394 | 0.001* |
| **Attitude 4: Do you consider that access to an outpatient cardiac rehabilitation center could be an added value in the country?**  **Likert scale 1 to 5** | 0.710 | 0.239 | 2.976 | 0.004* |
| **Attitude 5: Do you support the implementation of a home-based cardiac tele-rehabilitation program in Saudi Arabia?**  **Likert scale 1 to 5** | 0.428 | 0.195 | 2.197 | 0.030* |
| **Years of practice groups**  **<5 years compared to** |  |  |  |  |
| *5-10 years* | -0.125 | 0.457 | -0.274 | 0.785 |
| *11-15 years* | 0.553 | 0.434 | 1.274 | 0.206 |
| *16-20 years* | 0.520 | 0.539 | 0.964 | 0.338 |
| *>20 years* | 0.603 | 0.453 | 1.330 | 0.187 |
| **Workplace type**  **Public compared to** |  |  |  |  |
| *Private* | 0.951 | 0.456 | 2.085 | 0.040* |
| *Both* | 0.049 | 0.439 | 0.112 | 0.911 |
| **Workplace region**  **Central region compared to** |  |  |  |  |
| *Eastern region* | -0.346 | 0.477 | -0.726 | 0.469 |
| *Northern region* | -0.983 | 1.043 | -0.942 | 0.348 |
| *Other/Unknown* | -0.268 | 0.580 | -0.463 | 0.645 |
| *Southern region* | -0.094 | 0.520 | -0.181 | 0.857 |
| *Western region* | 0.386 | 0.383 | 1.006 | 0.317 |
| **Workplace Size**  **0-99 Beds compared to** |  |  |  |  |
| *100-299* | 0.195 | 0.371 | 0.526 | 0.600 |
| *300-499* | -0.687 | 0.384 | -1.787 | 0.077 |
| *500-999* | 0.357 | 0.462 | 0.773 | 0.441 |
| *>=1000* | -0.446 | 0.566 | -0.789 | 0.432 |
| **Manage PCI patients**  **No compared to Yes** | 0.963 | 0.471 | 2.046 | 0.043* |
| **Number of PCI patients seen per week**  **0-10 patients compared to** |  |  |  |  |
| *11-20* | -0.194 | 0.354 | -0.549 | 0.584 |
| *21-30* | -0.478 | 0.419 | -1.139 | 0.257 |
| *31-40* | -0.554 | 0.594 | -0.932 | 0.353 |
| *>40* | 0.106 | 0.463 | 0.229 | 0.820 |
| **Recommendation for post PCI patients**  ***Exercise a bit* versus** |  |  |  |  |
| *Do nothing, to be at rest versus* | 1.229 | 0.843 | 1.459 | 0.148 |
| *Quit smoking if they were smokers* | 0.188 | 0.380 | 0.494 | 0.622 |
| *See a therapist if they need mental health support* | 2.5623 | 1.380 | 1.857 | 0.066 |
| *Start to attend a cardiac rehabilitation program* | 1.329 | 0.415 | 3.206 | 0.002* |
| **Percentage of patients referred to cardiac rehabilitation**  **Nill compared to** |  |  |  |  |
| *Service is not available* | 0.576 | 0.529 | 1.089 | 0.279 |
| *Less than 50%* | 0.942 | 0.383 | 2.461 | 0.016* |
| *More than 50%* | 0.778 | 0.413 | 1.883 | 0.063* |
| *Unknown* | 0.381 | 0.619 | 0.615 | 0.540 |
| **Referred any patient to cardiac rehabilitation**  **Nill/Service not available compared to referred any patient**  ***(missing = 7)*** | 0.678 | 0.303 | 2.24 | 0.028* |
| **When cardiac rehabilitation should be prescribed for post PCI patients**  **Starting in the hospital settings versus** |  |  |  |  |
| *Directly after discharge in their first visit* | -0.077 | 0.345 | -0.223 | 0.824 |
| *4 weeks or more after their discharge* | -0.115 | 0.460 | -0.251 | 0.802 |
| *Would not prescribe it* | -0.740 | 0.410 | -1.805 | 0.074 |
| **How difficult is it to refer patients to cardiac rehabilitation in Saudia Arabia**  **Extremely difficult versus** |  |  |  |  |
| *Somewhat difficult* | 0.253 | 0.327 | 0.774 | 0.441 |
| *Neither easy nor difficult* | -0.075 | 0.440 | -0.17 | 0.865 |
| *Somewhat easy* | -0.304 | 0.596 | -0.509 | 0.612 |
| *Extremely easy* | 0.875 | 0.763 | 1.147 | 0.254 |
| **What barriers do you face to refer a patient to cardiac rehabilitation (multiple selection)** |  |  |  |  |
| *Lack of Services is a Barrier - No compared to Yes* | -0.039 | 0.320 | -0.122 | 0.903 |
| *Lack of Knowledge is a Barrier - No compared to Yes* | -0.660 | 0.307 | -2.152 | 0.034 * |
| *Lack of Local Systems is a Barrier - No compared to Yes* | -0.342 | 0.285 | -1.200 | 0.233 |
| *Patient Factors is a Barrier - No compared to Yes* | -0.262 | 0.302 | -0.870 | 0.387 |
| *Other Factors is a Barrier - No compared to Yes* | 0.031 | 0.568 | 0.054 | 0.957 |
| *No Barriers is a Barrier - No compared to Yes* | 0.032 | 0.482 | 0.065 | 0.948 |
| **Who should take the initiative to implement this kind of outpatient cardiac rehabilitation programs in Saudia Arabia (multiple selection)** |  |  |  |  |
| *All Care Providers - No compared to Yes* | -0.170 | 0.294 | -0.578 | 0.564 |
| *Ministry of Health - No compared to Yes* | -0.171 | 0.288 | -0.594 | 0.554 |
| *Policy Makers - No compared to Yes* | 0.451 | 0.289 | 1.561 | 0.122 |
| *Insurance Companies - No compared to Yes* | 0.093 | 0.288 | 0.321 | 0.749 |
| *Physicians - No compared to Yes* | 0.142 | 0.288 | 0.493 | 0.623 |

**PCI,** Percutaneous Coronary Intervention
