# Supplementary material for: Knowledge, attitudes, and practices of cardiac rehabilitation and barriers to referral among cardiologists in Saudi Arabia: A cross-sectional survey
Source: PLoS One. 2025 May 16;20(5):e0323694. doi: 10.1371/journal.pone.0323694 (PMC12083838; doi:10.1371/journal.pone.0323694)
Supplement: S2 Table — (DOCX) [file pone.0323694.s002.docx]

## **Supplementary Table 2: Practice Univariate Logistic Regression n=99 (missing=7)**

|  | **Estimate** | **Std. Error** | **z value** | **P** |
| --- | --- | --- | --- | --- |
| **Sex: Male compared to Female** | 0.406 | 0.704 | 0.0.576 | 0.565 |
| **Age category**  **<35 compared to** |  |  |  |  |
| *35-40* | 0.288 | 0.673 | 0.428 | 0.669 |
| *41-55* | -0.039 | 0.646 | -0.061 | 0.952 |
| *56-65* | -0.357 | 0.802 | -0.445 | 0.656 |
| *>65* | 14.873 | 1455.398 | 0.010 | 0.992 |
| **Nationality**  **Non-Saudi Arabian compared to Saudi Arabian** | 0.700 | 0.2850 | 1.704 | 0.088* |
| **Specialty Level**  **Fellow compared to** |  |  |  |  |
| *Consultant* | 0.159 | 0.579 | 0.275 | 0.783 |
| *Specialist* | 0.742 | 0.767 | 0.967 | 0.333 |
| **Education Location**  **Saudi Arabia compared to** |  |  |  |  |
| *Middle East/Africa Not Saudi Arabia* | -1.322 | 0.729 | -1.813 | 0.070* |
| *USA* | -0.852 | 0.865 | -0.985 | 0.325 |
| *Canada* | 0.182 | 0.694 | 0.263 | 0.793 |
| *Europe* | -0.734 | 0.763 | -0.962 | 0.336 |
| *South Asia* | 15.427 | 1696.734 | 0.009 | 0.993 |
| *Multiple* | -0.446 | 1.290 | -0.346 | 0.729 |
| *Unknown* | -0.852 | 0.676 | -1.260 | 0.208 |
| **Total Knowledge Score (Max range 2-10)** | 0.335 | 0.156 | 2.148 | 0.032* |
| **Attitude 1: Do you think that a patient who is stable post percutaneous coronary intervention procedure should be enrolled into a cardiac rehabilitation program?**  **Likert scale 1 to 5** | -0.217 | 0.290 | -0.747 | 0.455 |
| **Attitude 2: Do you think that cardiac rehabilitation in Saudi Arabia is effective?**  **Likert scale 1 to 5** | 0.506 | 0.221 | 2.288 | 0.022* |
| **Attitude 3: Do you think that your patients’ outcomes improved when they are enrolled in cardiac rehabilitation?**  **Likert scale 1 to 5** | 0.195 | 0.331 | 0.589 | 0.556 |
| **Attitude 4: Do you consider that access to an outpatient cardiac rehabilitation center could be an added value in the country?**  **Likert scale 1 to 5** | -0.296 | 0.398 | -0.745 | 0.456 |
| **Attitude 5: Do you support the implementation of a home-based cardiac tele-rehabilitation program in Saudi Arabia?**  **Likert scale 1 to 5** | -0.046 | 0.3085 | -0.148 | 0.882 |
| **Years of practice groups**  **<5 years compared to** |  |  |  |  |
| *5-10 years* | 0.156 | 0.683 | 0.228 | 0.819 |
| *11-15 years* | 0.030 | 0.654 | 0.046 | 0.964 |
| *16-20 years* | 0.780 | 0.939 | 0.830 | 0.406 |
| *>20 years* | 0.087 | 0.667 | 0.130 | 0.896 |
| **Workplace type**  **Public compared to** |  |  |  |  |
| *Private* | 0.827 | 0.825 | 1.002 | 0.317 |
| *Both* | 1.050 | 0.810 | 1.296 | 0.195 |
| **Workplace region**  **Central region compared to** |  |  |  |  |
| *Eastern region* | -0.767 | 0.719 | -1.067 | 0.286 |
| *Northern region* | -1.173 | 1.449 | -0.809 | 0.418 |
| *Other/Unknown* | -0.480 | 0.922 | -0.520 | 0.603 |
| *Southern region* | -2.271 | 0.876 | -2.593 | 0.010* |
| *Western region* | -0.480 | 0.592 | -0.810 | 0.418 |
| **Workplace region Central versus the others** | 0.898 | 0.440 | 2.043 | 0.041* |
| **Workplace Size**  **0-99 Beds compared to** |  |  |  |  |
| *100-299* | 0.124 | 0.610 | 0.204 | 0.839 |
| *300-499* | -0.259 | 0.627 | -0.412 | 0.680 |
| *500-999* | -0.887 | 0.698 | -1.271 | 0.204 |
| *1000* | 0.211 | 0.9319 | 0.227 | 0.821 |
| **Manage PCI patients**  **No compared to Yes** | -0.190 | 0.866 | -0.220 | 0.826 |
| **Number of PCI patients seen per week**  **0-10 patients compared to** |  |  |  |  |
| *11-20* | 0.095 | 0.553 | 0.172 | 0.863 |
| *21-30* | -0.955 | 0.631 | -1.512 | 0.131 |
| *31-40* | 0.095 | 0.912 | 0.105 | 0.917 |
| *>40* | 0.383 | 0.751 | 0.510 | 0.610 |
| **Recommendation for post PCI patients**  **Exercise a bit versus** |  |  |  |  |
| *Do nothing, to be at rest* | 15.1 | 1385 | 0.011 | 0.991 |
| *Quit smoking if they were smokers* | -1.430 | 0.828 | -1.727 | 0.084 |
| *See a therapist if they need mental health support* | 15.1 | 2399 | 0.006 | 0.995 |
| *Start to attend a cardiac rehabilitation program* | 0.368 | 0.949 | 0.388 | 0.698 |
| **When cardiac rehabilitation should be prescribed for post PCI patients**  **Starting in the hospital settings versus** |  |  |  |  |
| *Directly after discharge in their first visit* | 0.767 | 0.700 | 1.096 | 0.273 |
| *4 weeks or more after their discharge* | -0.486 | 0.780 | -0.622 | 0.534 |
| *Would not prescribe it* | -3.818 | 1.087 | -3.514 | <0.001* |
| **How difficult is it to refer patients to cardiac rehabilitation in Saudia Arabia**  **Extremely difficult versus** |  |  |  |  |
| *Somewhat difficult* | 1.298 | 0.506 | 2.566 | 0.010* |
| *Neither easy nor difficult* | 1.954 | 0.832 | 2.349 | 0.019* |
| *Somewhat easy* | 1.954 | 1.129 | 1.730 | 0.084* |
| *Extremely easy* | 16.729 | 1199 | 0.014 | 0.989 |
| **What barriers do you face to refer a patient to cardiac rehabilitation (multiple selection)** |  |  |  |  |
| *Lack of Services is a Barrier - No compared to Yes* | -0.971 | 0.552 | 1.759 | 0.079* |
| *Lack of Knowledge is a Barrier - No compared to Yes* | 0.419 | 0.504 | 0.830 | 0.406 |
| *Lack of Local Systems is a Barrier - No compared to Yes* | -0.204 | 0.436 | -0.469 | 0.639 |
| *Patient Factors is a Barrier - No compared to Yes* | 0.818 | 0.496 | 1.649 | 0.099* |
| *Other Factors is a Barrier - No compared to Yes* | 0.190 | 0.866 | 0.220 | 0.826 |
| *No Barriers is a Barrier - No compared to Yes* | 1.436 | 1.084 | 1.325 | 0.185 |
| **Who should take the initiative to implement this kind of outpatient cardiac rehabilitation programs in Saudia Arabia (multiple selection)** |  |  |  |  |
| *All Care Providers - No compared to Yes* | 0.284 | 0.439 | 0.647 | 0.518 |
| *Ministry of Health - No compared to Yes* | -0.739 | 0.447 | -1.652 | 0.099* |
| *Policy Makers - No compared to Yes* | -0.410 | 0.438 | -0.938 | 0.348 |
| *Insurance Companies - No compared to Yes* | 0.035 | 0.435 | 0.081 | 0.936 |
| *Physicians - No compared to Yes* | 0.785 | 0.440 | 1.784 | 0.075* |

**PCI,** Percutaneous Coronary Intervention
