# Supplementary material for: Knowledge, attitudes, and practices of cardiac rehabilitation and barriers to referral among cardiologists in Saudi Arabia: A cross-sectional survey
Source: PLoS One. 2025 May 16;20(5):e0323694. doi: 10.1371/journal.pone.0323694 (PMC12083838; doi:10.1371/journal.pone.0323694)
Supplement: Attachment — Submitted filename: Response to Reviewers.docx [file pone.0323694.s005.docx]

**Dear reviewers and editors,**

**We extend our gratitude for your valuable suggestions, which have significantly enhanced and strengthened our manuscript.**

**Response to Review – 25/03/2025**

| **Reviewer Comments** | **Response** | **Location of Change (Page # & continuous line #)** |
| --- | --- | --- |
| **Reviewer 1** |  |  |
| 1. The discussion should further explore the reasons behind regional disparities in referral rates, considering infrastructure, policy differences, and healthcare accessibility. | Thank you for your valuable feedback. We have addressed this comment by further exploring the reasons behind regional disparities in referral rates, considering infrastructure, policy differences, and healthcare accessibility in the revised discussion.  Please Note :We have added more references in the reference list.  **Direct quotes of changes from the revised manuscript:**  *“The delivery of CR in KSA is significantly hindered by a lack of integration across various levels of cardiac care, stemming from the nation’s fragmented healthcare system. The healthcare system in KSA is divided into two main sectors: the governmental sector, which provides free services to citizens, and the private sector, which operates on a fee-for-service basis. The Ministry of Health directly manages and funds 60% of governmental healthcare institutions, while the remaining 40% fall under the jurisdiction of various other agencies, including military and educational institutions. This fragmentation is evident in our findings, where 74.5% of physicians reported difficulties in issuing CR referrals, along with significant delays in referrals reaching eligible patients, who struggle to navigate the healthcare system [32].*  *The situation is further complicated by poor coordination and communication between cardiologists in provinces with limited cardiology care and CR providers, who are primarily concentrated in major cardiac centers within only two regions (Central and Eastern). Interviews with directors of cardiac centers and CR coordinators in KSA provided valuable insights into the factors contributing to the delayed expansion and uneven distribution of CR services across the country [36]. The qualitative analysis revealed widespread recognition of CR and its benefits among healthcare leaders, accompanied by significant concerns regarding the inadequacy of the current infrastructure to meet national demand [36]. According to policymakers, this challenge is primarily attributed to limited resources, particularly insufficient funding and a shortage of CR-trained personnel [36].*  *.”* | **Page 15-16, Line 372-391** |
| 2. The study's reliance on convenience sampling introduces potential selection bias, which should be discussed in more detail along with recommendations for future randomized studies. | Thank you for your insightful comment. We have addressed this concern by discussing the potential selection bias introduced by the convenience sampling strategy in the limitations section and by recommending future randomized trials in the conclusion.  **Direct quotes of changes from the revised manuscript:**  *“The cross-sectional design, which relied on a convenience sampling strategy, limited the recruitment base and restricted data collection to a short period, potentially introducing selection bias.”*  *“Future research should include comprehensive randomized trials involving all levels of cardiology specialists in KSA, including graduate medical students enrolled in cardiology fellowships, to evaluate their KAP regarding CR.”* | **Page 18 Line 454-456**  **Page 19 Line 481-483** |
| 3. The section on home-based cardiac rehabilitation (HBCR) should provide more details on practical implementation in Saudi Arabia, considering infrastructure, patient preferences, and feasibility. | Thank you for your feedback. We have addressed this comment by discussing the feasibility and suitability of HBCR in Saudi Arabia, as demonstrated by a recent three-arm RCT. The revised section highlights how HBCR effectively overcomes accessibility barriers, including geographic constraints, transportation challenges, and financial burdens. The revised section highlights key challenges, including the need for telemonitoring technologies, trained personnel, home-based medical equipment, and factors influencing patient acceptance, such as digital literacy and cultural aspects. Additionally, we emphasize the need for strong policy support, viable financial models, and ongoing professional training to ensure its successful and sustainable implementation in Saudi Arabia.  Please Note :We have added more references in the reference list.  **Direct quotes of changes from the revised manuscript:**  *“The home-based model has proven effective in overcoming several barriers to CR accessibility, addressing challenges such as limited facilities, geographic constraints, transportation difficulties, financial burdens, and scheduling conflicts [39]. Globally, HBCR has been shown to be as safe and effective as traditional hospital-based CR for patients at low to moderate risk following revascularization, offering comparable costs while enhancing participation and adherence rates [40,41]. In KSA, a recent three-arm RCT—the first conducted in the country and the Gulf region—demonstrated the feasibility and suitability of HBCR in the local context, showing it to be as effective as outpatient CR and superior in maintaining post-intervention improvements in patients with CHD following coronary artery bypass grafting [42]. Additionally, patient preferences and acceptance must be considered, particularly in terms of digital literacy, cultural factors, and willingness to engage in remote rehabilitation. The successful and sustainable expansion of HBCR programs in KSA further depends on strong policy support, the development of viable financial models, and ongoing professional training and education [36]. Despite its potential, successful implementation of HBCR in KSA also requires addressing infrastructure challenges, such as the availability of telemonitoring technologies, trained personnel, and home-based medical equipment.”* | **Page 16-17 Line 413-429** |
